# An anxiogenic drug, FG 7142, induced an increase in mRNA of Btg2 and Adamts1 in the hippocampus of adult mice

**DOI:** 10.1186/1744-9081-8-43

**Published:** 2012-08-22

**Authors:** Akeo Kurumaji, Toru Nishikawa

**Affiliations:** 1 Section of Psychiatry and Behavioral Sciences, Tokyo Medical and Dental University Graduate School, 1-5-45 Yushima, Bunkyo-ku, Tokyo 113-8519, Japan

**Keywords:** Anxiety, Hippocampus, FG7142, Flumazenil, Btg2, Adamts1, RT-PCR, GABA_A_ receptors, Dentate gyrus, Development

## Abstract

**Background:**

Anxiety and stress-related disorders are among the most common psychiatric disorders. The hippocampus is a crucial brain area involved in the neural circuits of the pathophysiology of anxiety and stress-related disorders, and GABA is one of most important neurotransmitters related to these disorders. An anxiogenic drug and a pharmacological stressor, FG7142 (N-methyl-ß-carboline-3-carboxamide), produces anxiety in humans and experimental animals, acting at the benzodiazepine sites of the GABA_A_ receptors as a partial inverse agonist. This drug as well as immobilization stress produced an increased mRNA in a number of genes, e.g., Btg2 and Adamsts1, in the cortex of rodents. The present study was carried out to clarify the effect of the anxiogenic drug on the gene expressions in the hippocampus and to obtain a new insight into the GABAergic system involved in the pathophysiology of the disorders.

**Method:**

We examined the effects of FG7142 on the gene expression of Btg2 and Adamts1 in the hippocampus of mice using a quantitative RT-PCR method as well as an in situ hybridization method.

**Results:**

The intraperitoneal administration of FG7142 at a dose of 20 mg/kg, but not 10 mg/kg, induced a statistically significant increase in the hippocampal mRNA of both genes in adult mice (postnatal days 56), being blocked by co-administrations of flumazenil (twice of 10 mg/kg, i.p.), an antagonist at the benzodiazepine binding site, while FG7142 failed to produce any change in the gene expressions in infant mice (postnatal days 8). In addition, the in situ hybridization experiment demonstrated an upregulation of the gene expressions restricted to the dentate gyrus of the hippocampus in adult mice.

**Conclusions:**

The present study suggests a functional coupling between the GABAergic system and the transcriptional regulation of the two genes (Btg2 and Adamsts1) in the hippocampus of adult mice, which may play a role in the brain function related to anxiety and stress such as memory of fear.

## Background

FG7142 (N-methyl-ß-carboline-3-carboxamide), an anxiogenic drug and/or a pharmacological stressor, produces anxiety in humans [1], rhesus monkeys [2] and rodents [3,4] along some physiological and neurochemical changes related to anxiety and stress [5]. Both FG7142 and stress, such as foot-shock and restraint stress, similarly produce an increased release of dopamine [6,7], noradrenaline [8,9], and glutamate in the cerebral cortex of rodents [10]. In addition, FG7142 produced an increased Fos-like immunoreactivity in the cortex of rats along with some brain areas associated with neuronal circuits mediating anxiety and stress-responses [11-14]. FG 7142 acts at the benzodiazepine sites of the GABA_A_ receptors as a partial inverse agonist and allosterically inhibits the ability of GABA to bind to and activate the receptors, which can be blocked by a benzodiazepine antagonist, e.g., flumazenil (Ro 15–1788) [15]. We have previously reported an increase in the mRNA of a number of genes, e.g., Btg2 and Adamts1, in the cortex of mice as a consequence of the modulation of the GABA_A_ receptors by the systemic administration of FG7142 [16]. In addition, exposure to a novel circumstance induced an acute increase in the gene expression of Btg2 in the hippocampus [17].

The hippocampus is known to be a crucial structure for the formation of certain types of memory, such as episodic memory and spatial memory [18]. Several lines of evidence have led to the view that the hippocampus also plays an important role in the memory of fear, an emotional state induced by a frightening event or stress [19]. In addition, new neurons are continuously added to the hippocampus throughout adult life in various species including humans [20]. The adult hippocampal neurons originate from a radial glia-like precursor cell (Type 1) in the subgranular zone of the dentate gyrus through a number of intermediate cell types (types 2 and 3). After an early postmitotic maturation stage, associated with dendrite and axon elongation and selective survival, the newborn granule cells fully integrate into the DG circuitry as well as the functions of the hippocampus [21].

Btg2 is an antiproliferative gene involved in the control of the cell cycle progression, specifically in the G1 to S transition [22,23]. Btg2 is specifically expressed in neurogenic precursor cells but not in postmitotic neurons during embryonic neurogenesis [24,25]. During adult hippocampal neurogenesis in newly generated cells, Btg2 was absent from the radial glia-like putative stem cells (type-1), first appeared in the transient amplifying progenitor cells (type-2 and −3 cells), was expressed again in maturing neurons, and finally decreased in mature granule cells. It is likely that the Btg2 expression in maturing granule cells might be linked to integration into the already existing neuronal networks and is subject to the inputs of various neurotransmitter systems during the maturation. Moreover, the involvement of Btg2 during fear conditioning was suggested by a behavioral experiment using the knock-out mice [26] and a genome-wide association study using a trait loci mapping method [27]. Adamts1 is the first member of ADAMTS (a disintegrin and metalloprotease with thrombospondin motifs) [28] and has been shown to display anti-angiogenic properties [29] and a role in the turnover of the extracellular matrix in the central nervous system [30]. It has been reported that the gene expression of Adamts1 was increased by an insult of ischemia in the hippocampus [31] and cerebral cortex [32,33] of rodents.

In the present study, we examined the effects of FG7142 on the gene expression of Btg2 and Adamsts1 in the hippocampus using a RT-PCR method and carried out an in situ hybridization experiment in order to obtain anatomically detailed information on the drug-effect in the brain area.

## Methods

### Animals and chemicals

The present animal experiments were performed in strict accordance with the guidelines of the Tokyo Medical and Dental University and were approved by the Animal Investigation Committee. Male C57BL mice (Japan Clea Laboratories, Japan) at postnatal days (PND) 56 (body weights; 20–23 g) or 8 (body weights; 3.5-5.5 g) were used. The mice were kept at 24.0 ± 0.5°C in a humidity-controlled room under a 12-h light–dark cycle (lights on at 8:00 AM) with free access to food and water. The animal experiments were conducted during the light cycle.

FG7142 (N-methyl-ß-carboline-3-carboxamide)(TOCRIS, Bristol, UK) and flumazenil (ethyl-8-fluoro-5,6-dihydro-5-methyl-6-oxo-4 H-imidazo[1,5-a][1,4] benzodiazepine-3-carboxylate) [a gift from the Astellas Pharmaceutical Company (Tokyo, Japan)] were dissolved in saline/40% 2-hydroxypropyl-ß-cyclodextrin (Nacalai Tesque, Kyoto, Japan). FG 7142 was injected intraperitoneally in a volume of 10 ml/kg (PND 8) or in a volume of 5 ml/kg (PND 56). The control animals received only the vehicle. Flumazenil (10 mg/5ml/kg) was subcutaneously injected in the mice twice at 15 min before and 20 min after the injection of FG7142, taking into account the relatively short action of the drug. Consequently, the experiment consisted of four groups of animals, namely the vehicle + vehicle, vehicle + FG7142, flumazenil + vehicle and flumazenil + FG7142. No animals exhibited seizures in the behavioral monitoring throughout the experiments.

### Extract of total RNA

The mice were killed by cervical dislocation one hour later after the administration of FG7142. Both sides of the hippocampus were rapidly removed in the cold, frozen in liquid nitrogen, and stored at −80°C prior to use. Each frozen cortical tissue section was homogenized using a Polytron Homogenizer (Kinematica AG, Littau/Luzern, Switzerland) at 24,000 rpm for 10 sec, and its total RNA was extracted using the Quiagen Rneasy Midi System (Quiagen, Valencia, CA, USA).

### Quantitative RT-PCR

The RNA sample was treated with RNase-free DNase I (Invitrogen, Carlsbad, CA, USA) to remove any contaminating genomic DNA. The single-stranded cDNA was then synthesized from 1 μg of the DNase I-treated neocortical RNA using a SuperScript Preamplification system (Invitrogen, Carlsbad, CA, USA). The remaining RNAs were digested using Ribonuclease H (Invitrogen, Carlsbad, CA, USA), and the resulting cDNA suspended in 10 volumes of TE buffer (10 mM Tris–HCl, pH 8.0, and 1 mM EDTA) was used for the quantitative RT-PCR analysis described below.

Five μL of a diluted sample of each first strand product was amplified using 2μL of LightCycler FastStart DNA Master SYBER Green I (Roche Diagnostics, Mannheim, Germany) [34] and a pair of primers (Table 1) at the final concentration of 0.5 μM each and MgCl_2_ at the final concentration of 3 mM. PCR was performed in a total volume of 20 μL for 10 min at 95°C and 40 cycles of 15 s at 95°C, 5 s at 65°C and 10s at 72°C. The melting curve analysis was done by continuous acquisition from 65°C to 95°C with a temperature transition rate of 0.1°C/sec. In each assay, standard curves were generated from four increasing amounts of the pooled cDNA templates of equal volumes of the individual samples. The results were automatically calculated using the respective standard curves by the LightCycler analysis software version 3.5. Amplification of the single product in the RT-PCR was confirmed by monitoring the melting curve and by agarose gel electrophoresis. Expression of the glyceraldehyde-3-phosphate dehydrogenase (Gapdh) was determined as the internal standard for each sample, because the Gapdh gene has been reported to be a housekeeping gene that is constantly expressed in neural tissues [35]. The ratios of the results of the mRNAs of each gene to those of the Gapdh mRNAs were used for the statistical comparison as the normalized values of the mRNA of each gene. 

**Table 1:** Primer sequences for the semi-quantitative RT-PCR

**Official Symbol of Gene** **Gene name**	**Gene Bank ID**	**Bases spanned**	**Forward primer (5' to 3')**	**Reverse primer (5' to 3')**
**Gapdh**	NM_001001303.	211-439	CGGCAAATTCAACGGCACAGTCAA	TGGGGGCATCGGCAGAAGG
Glyceraldehyde-3-phosphate dehydrogenase				
**Btg2**	NM_007570	479-597	CC CCCCGGTGGCTGCCTCCTATG	GGGTCGGGTGGCTCCTATCTA
B-cell translocation gene 2				
**Adamts 1**	NM_009621.	550-896	GGCGCCCCACGGAGGAAG	AGGCGCTGGCTGAATGAAGAAC
A disintegrin-like and metalloprotease with thrombospondin type 1				

### In situ hybridization

In situ hybridization was performed as previously described [36,37]. Anaesthetized male mice at post-natal day 56 were fixed by perfusion with tissue fixative (GenoStaff, Tokyo, Japan) and the whole brain was embedded in paraffin. A 410-bp DNA fragment corresponding to the nucleotide position 893–1302 of the mouse Btg2 cDNA (GenBank accession number NM_007570) or a 465-bp DNA fragment corresponding to the nucleotide position 75–539 of the mouse Adamts1 cDNA (GenBank accession number NM_009621) was subcloned into the pBluescript II KS(+) vector (Stratagene, La Jolla, CA, USA). Digoxigenin (DIG)-labeled single-stranded riboprobes (antisense and sense as the control) were prepared by in vitro transcription using the T7 or T3 RNA polymerase (DIG northern starter kit, Roche, Nonnenberg, Germany). Hybridization was performed with the DIG-labeled RNA probes at 60°C for 18 h. The bound label was detected using NBT-BCIP, an alkaline phosphatase color substrate, and tissue slides were counterstained with Kernechtrot stain solution (Muto Pure Chemicals, Tokyo, Japan).

### Statistical analysis

Data are expressed as a percentage of the control and are mean ± SEM. Differences between two groups were tested with the two-tailed Student’s *t*-test. Differences between more than two groups were tested with one-ANOVA followed by the post hoc Dunnett’s test or two-way ANOVA followed by the Sheffe’s test.

## Results

**Figure 1:**
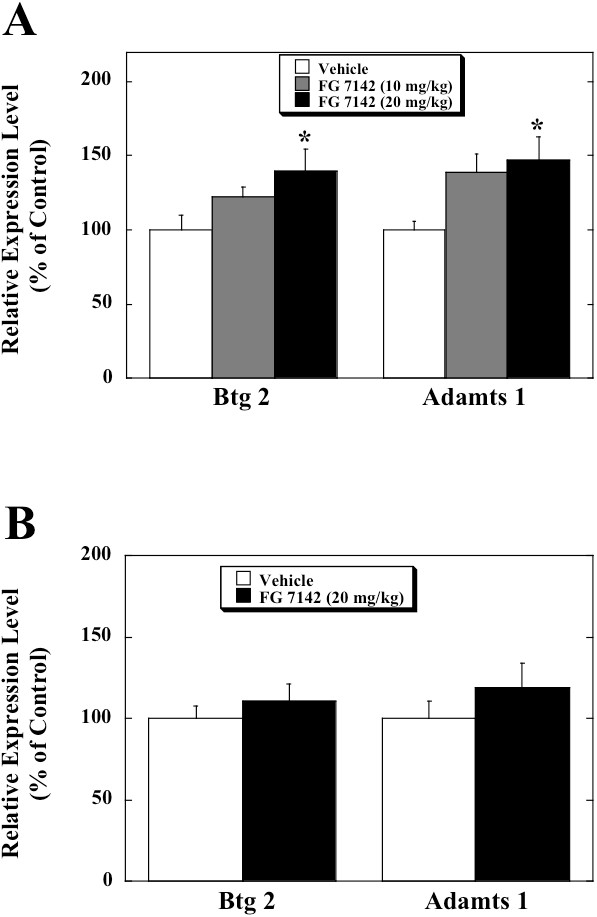
**Results of RT-PCR after treatment with FG 7142 in the hippocampus of (A) adult and (B) neonatal mice. **FG 7142 (10 or 20 mg/kg) or the vehicle was intraperitoneally injected into the adult (postnatal days 56) and neonatal (postnatal days 8) mice. The mice were sacrificed at one hour after the administration. (**A**) The data of adult mice are presented as a percentage of the mean value of the vehicle-treated mice and are mean ± SEM obtained from 6 or 7 animals. The statistical analysis was carried out by one-way ANOVA followed by the post hoc Dunnett’s test. * p < 0.05 compared to the vehicle-treated controls. (**B**) The data of neonatal mice are presented as a percentage of the mean value of the vehicle-treated mice and are mean ± SEM obtained from 9 or 10 animals.

**Figure 2:**
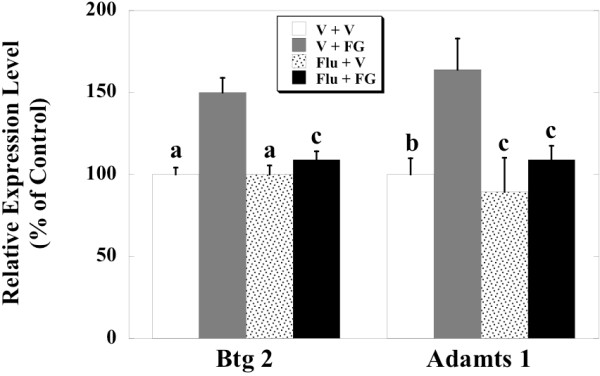
**Results of RT-PCR in the hippocampus of adult mice after treatment with flumazenil and FG 7142. **FG 7142 (20 mg/kg) or the vehicle was intraperitoneally injected into the adult mice (postnatal days 56) one hour before their sacrifice. Flumazenil (10 mg/kg) or the vehicle was subcutaneously injected into the mice twice, at 15 minutes before and 20 minutes after the administration of FG 7142 or the vehicle. Thus, the experiment consisted of four groups of animals, namely the vehicle + vehicle (V + V), vehicle + FG 7142 (V + FG), flumazenil + vehicle (Flu + V) and flumazenil + FG 7142 (Flu + FG). The data are expressed as a percentage of the mean value of the vehicle and vehicle-treated mice (V + V) and are mean ± SEM obtained from 6 or 7 animals. The statistical analysis was carried out by two-way ANOVA followed by the post hoc Sheffe’s test. a; p < 0.001 vs Vehicle + FG 7142, b; p < 0.01 vs Vehicle + FG 7142, c; p < 0.05 vs Vehicle + FG 7142.

The systemic injection of FG 7142 at a dose of 20 mg/kg induced a statistically significant increase in the mRNA of Btg2 [F (16,2) = 5.048, p < 0.05] as well as Adamts1 [F (2, 18) = 4.35, p < 0.05] in the hippocampus of the mice (Figure 1A). In the infant mice, however, there were no changes in the mRNA of either gene in the hippocampus after treatment with FG 7142 (20 mg/kg, i.p.) (Figure 1B). We examined the effects of co-administrations of flumazenil on the FG 7142-induced increases in the mRNAs of the genes (Figure 2). Two-way ANOVA revealed a significant effect of treatment with FG 7142 [Btg2: F (1,24) = 8.005, p < 0.01; Adamts1: F (1,24) = 7.925, p < 0.01] and a significant effect of treatment with flumazenil [Btg2: F (1,24) = 16.238, p < 0.001; Adamts1: F (1,24) = 12.897, p < 0.01] on the mRNA of the both genes. The analysis revealed a significant effect of the interaction of FG 7142 x flumazenil on the mRNA of Btg2 [F (1,24) = 8.169, p < 0.01] but not on the Adamts1 [F (1,25) = 3.619]. Treatment with FG 7142 failed to produce a statistically significant change in the mRNA expression levels of both genes in mice receiving the co-administrations of the benzodiazepine antagonist, while the administration of FG 7142 induced a statistically significant increase in the mRNA levels of the genes in mice injected with the vehicle of the antagonist. In addition, there was no difference between the hippocampal levels of the genes of mice treated with the vehicle of flumazenil and the vehicle of FG7142 and those of mice treated with flumazenil and the vehicle of FG7142.

**Figure 3:**
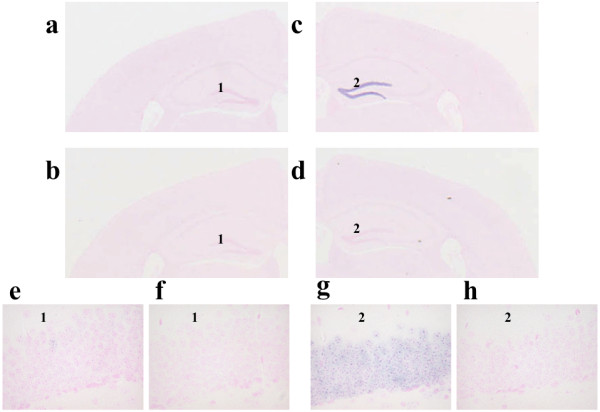
**In situ hybridization histochemistry of Btg2 in the hippocampus of adult mice after treatment with FG 7142. **The coronal section at the hippocampus level of the adult (postnatal days 56) mouse brain was hybridized with the anti-sense and sense strands of Btg2. The RNA signals are detected by the DIG system as shown in the purple color, and the cell nuclei are counterstained with Kernechtrot solution as shown in pink. **a **and **e**; vehicle-treated mice hybridized with the anti-sense probe, **b **and **f**; vehicle-treated mice hybridized with the sense probe, **c **and **g**; FG 7142-treated mice hybridized with the anti-sense probe, **d **and **h**; FG 7142-treated mice hybridized with the sense probe.

**Figure 4:**
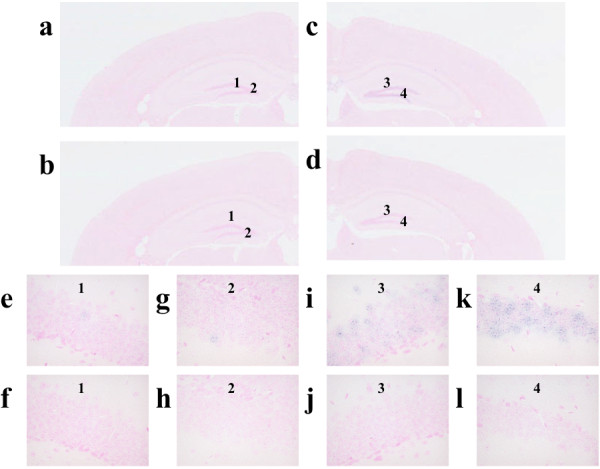
**In situ hybridization histochemistry of Adamts1 in the hippocampus of adult mice after treatment with FG 7142. **The coronal section at the hippocampus level of the adult (postnatal days 56) mouse brain was hybridized with the anti-sense and sense strands of Adamts1. The RNA signals are detected by the DIG system as shown in the purple color, and the cell nuclei are counterstained with Kernechtrot solution as shown in pink. **a**, **e **and **g**; vehicle-treated mice hybridized with the anti-sense probe, **b**, **f **and **h**; vehicle-treated mice hybridized with the sense probe, **c**, **i **and **k**; FG 7142-treated mice hybridized with the anti-sense probe, **d**, **j **and **l**; FG 7142-treated mice hybridized with the sense probe.

A prominent increase in the Btg2 mRNA signals with the DIG-labeled anti-sense RNA probe was observed in the molecular layer of the dentate gyrus of mice receiving the injection of 20 mg/kg of FG 7142, while there were sparse signals of the mRNA of Btg2 in the brain area of mice after the administration of the vehicle (Figure 3). In addition, treatment with FG 7142 induced an increase in the number of cells showing the Adamts1 signals detected by the anti-sense RNA probe in the molecular layer of the dentate gyrus, compared to those of mice receiving the vehicle (Figure 4).

## Discussion

FG7142 has been shown to induce anxiety-related behavioral and physiological responses in a variety of experimental paradigms across numerous mammalian species including humans [1-5] and to activate an anxiety-related neural network interacting [13,14] with other systems of neurotransmitters such as monoamines [6-9] and excitatory amino acid [10]. The anxiogenic effect of FG7142 on rodents assessed by the elevated plus-maze was demonstrated at doses between 10 and 100 mg/kg [3-5,15]. The RT-PCR experiments demonstrated that the mRNAs of Btg2 and Adamsts1 in the hippocampus of adult mice were increased by the systemic administration of FG7142 at a dose of 20 mg/kg, but not of 10 mg/kg. Because the drug failed to induce a significant increase in the hippocampal mRNAs in adult mice receiving the co-administrations of flumazenil, an antagonist at the benzodiazepine binding sites within the GABA_A_ receptors, it is postulated that the effects of FG7142 on the gene expression can be mediated via action of the drug as a partial inverse agonist at the benzodiazepine site.

The experiments of in situ hybridization clearly demonstrated that the FG7142-induced increases in the gene expression of the genes were confined to the dentate gyrus of the hippocampus, whereas it has been established that the benzodiazepine sites show a high density during the hippocampal formation including the dentate gyrus as well as Ammon’s horn [38]. Atack et al. reported that FG7142 had a relatively higher affinity for the α1 subunit than the other α subunits at the recombinant GABA_A_ receptors [15]. In addition, an abundant localization of the α1 subunit in the dentate gyrus was similarly demonstrated by immunocytochemical studies [39,40]. Thus, it is likely that a coupling between the GABA_A_ receptors and the transcriptional regulations of Btg2 and Adamsts1 is mainly associated with the subunit in the area.

The dentate gyrus is a key anatomical region of the excitatory trisynaptic pathway in the hippocampus. The axons of the layer II neurons in the entorhinal cortex project into the dentate gyrus through the perforant pathway. The dentate gyrus sends projections into the pyramidal cells in CA3 through mossy fibers. The CA3 pyramidal neurons relay information to CA1 pyramidal neurons through the Schaffer collateral. The CA1 pyramidal neurons send back-projections into the deep-layer neurons of the entorhinal cortex [21]. In addition, adult neurogenesis, originating from neural progenitor cells, is observed in the subgranular zone of the dentate gyrus. Neurons born in the subgranular zone differentiate and integrate into the local neural network as granule cells of the dentate gyrus by regulations of the GABAergic and glutamatergic synaptic inputs [21].

Btg2 is a transcriptional co-factor endowed with antiproliferative and prodifferentiative properties. During embryonic neurogenesis, the expression has been associated with the switch from proliferation to neurogenesis and lengthening of the cell cycle [41,42]. The expression of Btg2 has been detected in the dentate gyrus of the adult hippocampus, in type-2 progenitor cells and in differentiated neurons [43]. We have recently reported that exposure to a novel circumstance, a mild psychological stressor, induced an acute and short-lasting increase in the mRNA of Btg2 in the hippocampus of mice [17]. Btg2-null mice showed impairment in contextual fear conditioning, without any deficit in spatial memory [26]. In addition, a genome-wide association study of fear conditioning using a quantitative trait loci mapping method and bioinformatic analyses identified a number of candidate genes including Btg2 as well as subunits of GABA_A_ receptors, e.g., Gabra2 (GABA_A_ receptor, subunit α-2) and Gabra1b (GABA_A_ receptor, subunit β-1) [27]. Thus, the present study suggests that there may be a functional coupling between the GABA_A_ receptors and expression of the Btg2 gene in the discrete brain area and that the anxiogenic drug-induced gene expression in the matured neurons may play a role in the early stage of the molecular system responsive to anxiety and/or stress.

The present study also demonstrated the transcriptional activation of the Adamts1 gene by the partial inverse agonist of the benzodiazepine receptors in the hippocampus of adult mice, while the previous study showed no responses of the gene to the novelty stress in the hippocampus [17]. The effect of the novelty stress on the neural transmission of the GABA_A_ receptors may be different from that of the benzodiazepine receptors-related drug in magnitude and/or manner, as the brain responsive system to stress involves various neurotransmitters [5] and consists of neuronal circuits [11-14].

There were no changes in the hippocampal gene expressions of infant mice after treatment with FG7142 in the present study. We have previously shown age-dependent inductions of a number of genes by schizophrenomimetic drugs. The acute treatment with phencyclidine or methamphetamine significantly increased the neocortical expression of the genes (e.g., mrt1 [44], Cyr61 [45] and leiomodin2 [46]) at postnatal day 56, but not at postnatal day 8, suggesting that there was a relation with the developmentally regulated drug-induced abnormal behaviors. Physiological functions of the GABA_A_ receptors in the hippocampal neurons during the first postnatal week are significantly different from those of the adult brain, providing an excitatory rather than inhibitory drive due to a different Cl^-^ gradient [47]. Thus, further studies are required to identify the developmental age showing the crucial alteration in the induction of the Btg2 and Adamsts1 genes as well as behavioral alterations associated with anxiety after treatment with the anxiogenic drug.

## Conclusions

Because several lines of evidence indicate the involvement of GABA_A_ receptors in the function of the hippocampus associated with not only anxiety and fear but also memory and learning [18,19], the present study suggests that the transcriptional activation of Btg2 and Adamts1 followed by the anxiogenic drug-induced reduction in the transmission of the GABA_A_ receptors in the adult hippocampus may be involved in changes of the neural functions in the emotional states and memory of the experience. Consequently, further investigations are necessary to clarify the cascade of the molecular response including translations of the mRNAs as well as the consequence of physiological function in the hippocampus after the enhanced gene expressions.

## Abbreviations

Adamts1, A disintegrin-like and metalloprotease with thrombospondin type 1; ANOVA, Analysis of variance; Btg2, B-cell translocation gene 2; CA1 or 3, Cornu ammonis 1 or 3; cDNA, Complementary deoxyribonucleic acid; DIG, Digoxigenin; GABA, **G**amma-**a**mino**b**utyric **a**cid; Gabra2, GABA_A_ receptor, subunit α-2; Gabra1b, GABA_A_ receptor, subunit β-1; Gapdh, Glyceraldehyde-3-phosphate dehydrogenase; mRNA, Messenger ribonucleic acid; NBT-BCIP, Nitro blue tetrazolium- 5-bromo-4-chloro-3-indolyl-phosphate; PND, Postnatal days; RT-PCR, Reverse transcription polymerase chain reaction.

## Competing interests

The authors declare that they have no competing interests.

## Authors’ contributions

AK and TN designed the present study and collaborated on the preparation and submission of this paper. AK carried out almost all the procedures of the experiments. TN dissected the hippocampus of all of the mice after the administration of the drug. Both authors read and approved the final manuscript.
